# Smartphone acquisition as an inflection point in early adolescence digital exposome: relevance to mental health

**DOI:** 10.3389/frcha.2026.1940646

**Published:** 2026-09-30

**Authors:** Ziv Bren, Kate T. Tran, Ronald Clinton, Elina Visoki, Lani A. Tran, Nadine Michel, Tyler M. Moore, Samuel D. Pimentel, Ran Barzilay

**Affiliations:** 1 Department of Child and Adolescent Psychiatry and Behavioral Sciences, Children’s Hospital of Philadelphia (CHOP), Philadelphia, PA, United States; 2 Lifespan Brain Institute of CHOP and Penn Medicine, Philadelphia, PA, United States; 3 Perelman School of Medicine, University of Pennsylvania, Philadelphia, PA, United States; 4 Department of Psychiatry, Perelman School of Medicine, University of Pennsylvania, Philadelphia, PA, United States; 5 Department of Statistics, UC Berkeley, Berkeley, CA, United States

**Keywords:** adolescence, exposome, mental health, smartphones, social media

## Abstract

**Objective:**

Acquisition of the first smartphone represents a major developmental transition. However, evidence on its effects on adolescent health and behavior remains limited. We assessed associations between first smartphone acquisition around age 12 and changes in mental health, environmental and lifestyle (exposomic) factors, and examined potential pathways underlying these associations.

**Methods:**

We analyzed longitudinal data from 3,486 participants in the U.S. Adolescent Brain Cognitive Development (ABCD) Study who did not have a smartphone by age 12 (2-year assessment wave). We tested the association between smartphone acquisition (exposure) between age 12 to 13 (the 2- and 3-year assessments) and age 13 self-reported mental health (Brief Problem Monitor [BPM]) and exposomic outcomes including digital (screen time, social media, gaming, streaming, cyberbullying) and offline (sleep duration, school performance, exercise, bullying and reading) measures. Mixed-effects regression models accounted for sociodemographic, family, and developmental covariates. To reduce confounding, models additionally adjusted for pre-exposure measures of each outcome assessed at age 12, prior to smartphone acquisition. Mediation models examined indirect effects of smartphone acquisition on mental health through total screen time, social media account ownership, and bedtime media use.

**Results:**

Compared with youth who remained smartphone-free at age 13 (*n* = 1,940), smartphone acquisition (*n* = 1,546) was associated with worse BPM internalizing, attention, and total scores (all p's ≤ 0.003), more screen time, greater odds of having a social media account, less sleep, and more frequent bedtime use of social media, video games, and streaming (all p's < 0.001). Smartphone acquisition was not associated with differences in externalizing symptoms, academic performance, exercise, or bullying/cyberbullying (all p's > 0.05). Mediation analyses suggested indirect associations between smartphone acquisition and mental health through total screen time, social media account ownership, and bedtime media use (all indirect effects *p* ≤ 0.002); collectively, these factors accounted for most of the association, with social media use contributing the largest indirect effect.

**Conclusion:**

Smartphone acquisition is associated with several environmental and lifestyle changes in the following year, particularly around bedtime. These changes may represent mechanisms linking smartphone acquisition to mental health symptoms. Findings provide insight into potential behavioral pathways associated with smartphone acquisition and adolescent mental health and may inform future intervention studies.

## 1 Introduction

Smartphones have become a central and nearly universal feature of adolescents’ daily lives (1). By age 11, approximately half of American children own a mobile phone, and by ages 13–17, 95% report owning a smartphone (2). Unlike other screen devices, smartphones are portable, personal, continuously connected, and embedded in daily life, often serving as adolescents’ primary means of communication, information access, and social engagement. As a result, most youth now have access to a personal mobile device, often engaging with it for several hours each day (3). This widespread adoption reflects a fundamental shift in how adolescents communicate, access information, and participate in social life during a developmental period characterized by heightened sensitivity to environmental exposures and social feedback. At the same time, mental health conditions among U.S. adolescents have emerged as an increasingly important public health concern, with the prevalence of diagnosed conditions increasing from 15.0% in 2016 to 20.3% in 2023 (4). Our prior work using data from the Adolescent Brain Cognitive Development (ABCD) Study suggests that risk may arise from smartphone ownership itself in early adolescence, even before considering specific patterns of use. Specifically, we found that smartphone ownership at age 12 is associated with higher odds of depression, obesity, and insufficient sleep (5).

First time smartphone acquisition represents an entry into a persistently accessible digital environment that enables a range of downstream behaviors. Among these, increased screen time is one observable consequence and has raised concerns due to its links with reduced physical activity, attentional difficulties, and mental health outcomes (6–9). Additionally, bullying and cyberbullying have been consistently associated with adverse mental health outcomes, underscoring the importance of understanding how emerging digital exposures intersect with these risk factors (10, 11). Within the evolving landscape of adolescent digital engagement, social media use has received particular attention because it is a central venue for peer interaction (including potentially negative interactions such as cyberbullying) and engages key psychosocial processes, such as social comparison and validation-seeking, that are central to this developmental stage (12). Frequent or dysregulated engagement with social media platforms has been associated with internalizing symptoms such as anxiety and depression, although findings remain mixed (13, 14). In addition, adolescent smartphone use has emerged as a significant concern for sleep health (15, 16). The accessibility of smartphones, particularly during evening hours, may delay sleep onset through cognitive and emotional stimulation, exposure to blue light, and the disruption of consistent bedtime routines, as shown previously on general screen use behaviors (17). As a result, smartphone use may contribute to later bedtimes and shortened sleep duration, highlighting a potential pathway linking engagement with smartphone with health outcomes.

### 1.1 The digital exposome

Beyond individual behaviors such as screen time or social media use, emerging work conceptualizes digital engagement within a wider ‘digital exposome’ framework (18). The exposome refers to the totality of environmental and lifestyle exposures individuals experience across the life course and their cumulative effects on health (19). Building on this concept, the digital exposome captures the set of exposures arising from interactions with digital technologies, including device use patterns, platform features, online social environments, and their combined effects on development and well-being. Grounded in developmental and ecological perspectives on adolescent digital media use, the digital exposome framework situates digital exposures within social, family, and developmental contexts rather than treating them as isolated phenomena (20). Accordingly, smartphone acquisition may be understood not only as access to a device, but also as a developmental transition that reorganizes adolescents’ daily routines, relationships, and engagement with their surroundings. This framework underscores that digital experiences are not discrete isolated behaviors but a network of interconnected exposures operating through behavioral and psychosocial pathways with mental health implications, particularly in adolescence. Recent work by our group further supports this perspective by showing that aggregated ‘digital exposomic risk’ that aggregates multiple domains of digital engagement around social media use explains substantial variance in adolescent mental health beyond offline exposome and beyond general screen time (21).

### 1.2 Smartphone acquisition as a developmental transition

Despite growing interest in smartphone-related behaviors, most research has focused on patterns of use among youth who already own a smartphone, rather than on the transition from non-ownership to smartphone acquisition (22, 23). Importantly, the acquisition of a first smartphone is not a uniform developmental experience. The timing and circumstances of smartphone acquisition may vary according to family socioeconomic resources, parental monitoring practices, cultural and family norms regarding technology use, educational needs, and peer influences (24). These factors may shape both access to smartphones and the ways in which adolescents subsequently engage with digital environments. Smartphone acquisition may represent a key developmental inflection point, marking a shift from intermittent, shared access to digital technologies toward continuous, private, and largely autonomous engagement. From a digital exposome perspective, this transition may expand adolescents’ exposure to digital environments, altering daily routines, including sleep patterns, media use habits, and engagement with social media and other online platforms (16, 25, 26). Such changes may have downstream implications for mental health and broader functioning (27–29), reflecting a shift in adolescents’ cumulative exposures across digital and offline environments (Figure 1).

**Figure 1:**
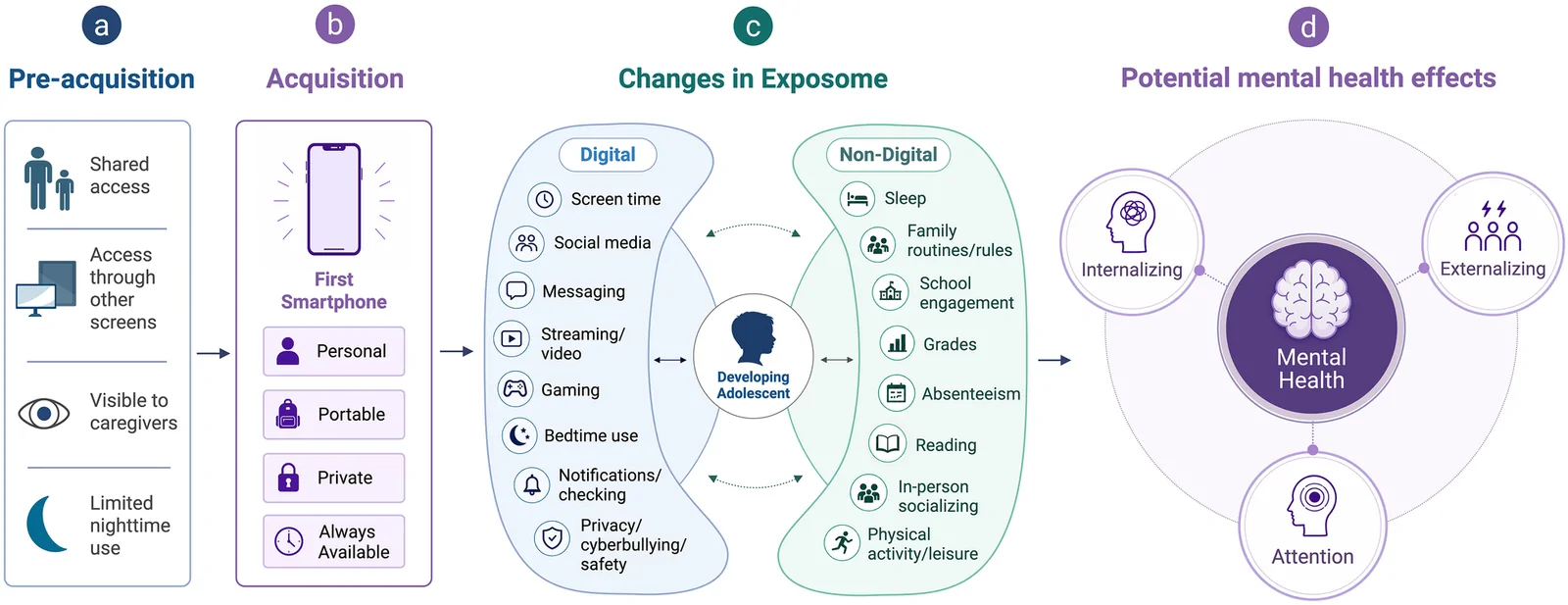
Conceptual model of first smartphone acquisition, changes in the adolescent exposome, and mental health outcomes **Legend:** This conceptual model illustrates the hypothesized pathways linking first smartphone acquisition during adolescence to changes in digital and non-digital exposomes that may subsequently influence mental health outcomes. Prior to smartphone acquisition **(a)**, access to digital technology may occur primarily through shared devices and other screens, with greater caregiver visibility and fewer opportunities for nighttime or private use. Following acquisition of a first personal smartphone **(b)**, adolescents gain continuous, portable, and more private access to digital technologies. Smartphone acquisition is hypothesized to alter multiple domains of the adolescent exposome **(c)**, Changes in digital exposures may include screen time, social media use, streaming video, gaming, bedtime smartphone use, notifications/checking behaviors, and privacy or cyberbullying-related experiences. Concurrent changes in non-digital exposures may include sleep, family routines, school engagement, academic performance, reading, in-person socialization, and physical activity or leisure activities. These digital and non-digital domains may interact dynamically during adolescent development. Collectively, these changes may contribute to mental health outcomes **(d)**, including internalizing symptoms, externalizing symptoms, and attention-related problems. The figure presents a conceptual framework for the hypothesized mechanisms examined in the study and is not intended to imply causal relationships among individual pathways.

### 1.3 Behavioral pathways linking smartphone use and mental health

Several mechanisms have been proposed to explain links between smartphone engagement and adolescent mental health, many of which map onto changes in daily behaviors and routines. One key pathway involves sleep-related behaviors: evening smartphone access may delay bedtimes, reduce sleep duration, and increase cognitive and emotional stimulation prior to sleep (25, 30). A second pathway involves attentional and cognitive processes. Smartphones enable rapid switching between activities and frequent interruptions, contributing to attentional fragmentation and reduced sustained focus, with potential downstream effects on academic engagement and performance (6, 31–33). A third pathway involves social and emotional processes, as constant connectivity increases exposure to peer interaction, social comparison, and feedback, which may shape emotional regulation and well-being (34, 35).

Critically, these mechanisms have largely been examined as correlates of smartphone use rather than as changes emerging from the acquisition of one's first smartphone. Framed within an exposomic perspective, smartphone acquisition may represent a shift that simultaneously alters multiple domains of experience—including digital engagement, sleep-related behaviors, and offline activities—introducing coordinated changes in adolescents’ online and offline exposome. Identifying these changes may also help pinpoint modifiable behaviors and exposures that could serve as targets for prevention efforts during a key developmental transition. This perspective motivates the current study, which tests whether such multidomain changes accompany smartphone acquisition and whether they account for associated differences in mental health.

### 1.4 The present study

In prior work, we found that smartphone ownership and age of smartphone acquisition were associated with several health outcomes in early adolescence, including depression, obesity and insufficient sleep (5, 36). However, these studies focused primarily on health outcomes rather than the behavioral and environmental changes accompanying smartphone acquisition. The mechanisms underlying these associations, and specifically whether they reflect shifts in adolescents’ exposome, remain unclear. The present study builds on this work by testing whether first smartphone acquisition represents an inflection point in the adolescent exposome and whether resulting changes in digital and offline exposures account for previously observed mental health differences. Leveraging longitudinal data, we focus on youth without smartphones at age 12 and examine changes that occur by age 13 among those who acquire a smartphone compared to those who do not. Critically, our design allows us to account for pre-acquisition levels of both exposomic factors and mental health, enabling us to isolate changes associated with smartphone acquisition rather than pre-existing differences between groups.

We pursue two primary aims. First, we test the hypothesis that smartphone acquisition is associated with changes across multiple domains of the exposome, including digital engagement, sleep-related behaviors, and broader health-related activities, and with different symptom domains of mental health. Second, we test whether exposomic changes may represent potential pathways linking smartphone acquisition and subsequent mental health outcomes. By focusing on acquisition as a developmental transition, this study aims to provide a more mechanistic and temporally grounded account of how smartphone access reshapes adolescents’ exposure landscape and contributes to adolescent mental health.

## 2 Methods

### 2.1 Participants

We analyzed data from the Adolescent Brain Cognitive Development (ABCD) Study, a large, ongoing, population-based longitudinal study of youth development conducted across 21 sites in the United States. Participants were recruited at ages 9–10 years using school-based sampling strategies designed to approximate the sociodemographic composition of the U.S. population and have been assessed annually from late childhood into adolescence using standardized protocols (37). The present analyses utilized data from the ABCD Study Data Release 5.1, which includes assessments conducted between 2016 and 2022. Smartphone ownership was first assessed at the 2-year follow-up; therefore, the analytic sample was restricted to participants who did not own a smartphone at that assessment wave and who also completed the subsequent 3-year follow-up assessment. This design enabled comparison of adolescents transitioning into smartphone ownership with those who remained without smartphones over the same developmental interval, while adjusting for baseline levels of outcomes measured prior to exposure, thereby improving causal interpretability. Outcomes were assessed at the 3-year follow-up. Covariates included both baseline measures assessed at the 2-year follow-up (prior to smartphone acquisition) and time-invariant demographic characteristics. Comparison of the participants included in this study with remaining ABCD participants who were not included (i.e., had already acquired a smartphone before the 2-year assessment) is provided in eSupplementary Table 1. All ABCD Study procedures were approved by the University of California, San Diego Institutional Review Board, and the present secondary analyses were determined to be exempt from review by the Children's Hospital of Philadelphia Institutional Review Board. Written informed consent was obtained from parents or legal guardians, and assent was obtained from participating youth. All analyses adhered to the Strengthening the Reporting of Observational Studies in Epidemiology (STROBE) guidelines (38).

### 2.2 Measures

Smartphone ownership was assessed via caregiver report. Caregivers were asked, ‘Does your child have their own cellphone?’ followed by an item querying device type. Participants with an iPhone or Android were considered as owning a smartphone. Our exposure measure of first smartphone ownership was defined as having no smartphone at the 2-year follow-up assessment and owning a smartphone at the 3-year follow-up assessment.

Mental health was assessed using the youth-reported Brief Problem Monitor (BPM) (39), a validated measure of youth emotional and behavioral functioning. We examined internalizing, externalizing, and attention subscales, along with the total problems score that represents overall symptom burden. All outcomes were treated as continuous variables, with standardized T-scores where higher scores indicate greater symptom severity (40).

Behavioral and environmental outcomes were assessed using youth self-report items and organized to capture online and offline contexts. Within the online environment, total screen time was derived from self-reported weekly screen use in hours and was treated as a continuous variable. Social media account ownership was assessed with the item, ‘Do you have a social media account?’ and coded as a binary variable indicating the presence of at least one account. Having a secret social media account was defined based on the item ‘Do you have a social media account that you keep secret from your parents?’ and was coded as a binary variable. Bedtime routines were assessed using past-week self-report items capturing media use while in bed before sleep. Participants were asked, ‘In the past week, how often did you do the following activities while already in bed before going to sleep?’ Separate items assessed the frequency of (1) social media use, (2) watching or streaming movies, videos, or TV shows, and (3) playing video games while in bed. Responses were coded on a 4-point scale reflecting the number of nights in the past week the activity occurred (1 = 0 nights, 2 = 1–2 nights, 3 = 3–4 nights, 4 = 5–7 nights). Each item was analyzed as a continuous measure of frequency. These behaviors were examined separately and were also combined into a composite indicator of bedtime entertainment media use, consisting of video gaming and watching or streaming movies, videos, or television, to capture non-social forms of pre-sleep media engagement. The composite was calculated by averaging the corresponding items. Cyberbullying exposure was assessed using the ABCD Cyberbully Questionnaire item ‘Have you ever been cyberbullied, where someone was trying on purpose to harm you or be mean to you online, in texts, or group texts, or on social media (like Instagram or Snapchat)?’ with responses coded as a binary variable indicating exposure (11). These online measures were included based on evidence that digital engagement and online peer interactions represent central mechanisms linking smartphone use to adolescent mental health (8, 11, 12, 17, 41).

Offline outcomes were assessed to capture adolescents’ broader social and functional experiences. In-person (offline) bullying involvement was measured using the Peer Experiences Questionnaire (42) and coded as a binary variable, with participants in the highest decile of scores classified as exposed. School functioning was assessed using two self-report items capturing attendance and performance. School attendance was measured with the item, ‘*How many days did you skip school without an excuse during the past 4 weeks?*’ and academic performance with the item, ‘*What grades did you receive in school last year?*’, with both variables treated as continuous indicators of academic functioning that may be influenced by smartphone ownership, as suggested by prior research in other populations (43). Physical activity was assessed with the item, ‘During the past 7 days, on how many days were you physically active for a total of at least 60 min per day?’ Responses were coded on an 8-point scale corresponding to the number of physically active days (0 = 0 days, 1 = 1 day, 2 = 2 days, 3 = 3 days, 4 = 4 days, 5 = 5 days, 6 = 6 days, and 7 = 7 days) and were treated as a continuous variable, given its relevance to adolescent health and potential sensitivity to changes in behavior associated with smartphone ownership (23). Bedtime reading was assessed using an item asking participants how often they engaged in reading while in bed before sleep during the past week. Responses were coded on a 4-point scale reflecting the number of nights in the past week the activity occurred (1 = 0 nights, 2 = 1–2 nights, 3 = 3–4 nights, and 4 = 5–7 nights) and were treated as a continuous variable. Because this measure was collected only at the 2-year follow-up (age 12) and was not available at the 3-year follow-up, it could not be incorporated into the longitudinal analyses of smartphone acquisition. Accordingly, bedtime reading was examined as an exploratory cross-sectional correlate of smartphone ownership at age 12 among ABCD participants assessed at the 2-year follow-up, comparing youth with and without smartphone ownership at that time point. Bedtime reading was conceptualized as a non-digital pre-sleep activity, providing a contrast to media-based bedtime behaviors (44).

Sleep duration was assessed using the youth version of the Munich Chronotype Questionnaire (45), based on reported sleep duration on school nights in hours and analyzed as a continuous variable. Sleep duration was included as a key health outcome and a potential pathway linking digital behaviors with emotional and behavioral functioning (15).

### 2.3 Covariates

All analyses adjusted for a comprehensive set of demographic, socioeconomic, and developmental covariates selected *a priori* based on their potential associations with both smartphone acquisition and study outcomes. In addition, to strengthen temporal ordering and reduce confounding, models incorporated pre-exposure measures of the outcome variables assessed at the 2-year follow-up (prior to smartphone acquisition). Specifically, each outcome measured at the 3-year follow-up was adjusted for its corresponding baseline value at the 2-year follow-up, thereby accounting for individual differences that predated smartphone acquisition.

Covariates further included age and sex assigned at birth, as well as household income, parental education, and parent-reported race and ethnicity. Race and ethnicity were included to account for potential confounding related to differences in access to smartphones and variation in behavioral and health outcomes across groups in the ABCD cohort (5).

Developmental and family context were captured through measures of pubertal status, categorized as pre-, early, mid-, late-, and post-pubertal, and parental monitoring, assessed as the mean score of five youth-reported items, each rated from 1 (Never) to 5 (Always) (46). To enhance the specificity of estimated associations to smartphone ownership, models additionally adjusted for ownership of other personal digital devices, including tablets, laptops, iPods, and smartwatches.

### 2.4 Statistical analysis

Descriptive statistics were computed to characterize the analytic sample and to compare adolescents who acquired a smartphone between the 2-year and 3-year follow-up assessment waves with those who did not. Distributions of continuous study variables were examined graphically and are presented in the Supplementary Materials (eSupplementary Figure 1). Although several variables exhibited departures from normality, continuous outcomes were retained in the primary analyses to preserve the full variability of the measures and to facilitate consistency and clinical interpretation of effect estimates. To assess the robustness of the findings to outcome distribution and alternative parameterizations, we conducted sensitivity analyses in which these outcomes were dichotomized using clinically or empirically meaningful thresholds when available and median splits otherwise. Specifically, BPM total problem, externalizing, attention, and internalizing scores were dichotomized at ≥65; bedtime activity variables were categorized as 0 nights versus ≥1 night in the past week; and bullying was defined using the previously established 90th-percentile threshold. The remaining continuous outcomes were dichotomized at their respective medians.

Group differences in continuous variables were evaluated using nonparametric tests to reduce reliance on distributional assumptions. Specifically, the Wilcoxon rank-sum test was used for comparisons between two groups, and the Kruskal–Wallis test was used when more than two groups were compared. Differences in categorical variables were assessed using Pearson's chi-square test, with Fisher's exact test used when appropriate. All statistical tests were two-sided, and a *p*-value <0.05 was considered statistically significant.

Multivariable analyses focused on two primary aims: (1) to characterize changes in adolescents’ digital and offline exposome associated with smartphone acquisition, and (2) to evaluate whether these changes accounted for associations between smartphone acquisition and subsequent mental health through mediation analyses.

Mixed-effects regression models were used to examine associations between smartphone acquisition and changes in mental health and across domains of the exposome, accounting for clustering within the ABCD study design. Continuous outcomes, including BPM T-scores, sleep duration, screen time, school attendance, and academic performance, were analyzed using linear mixed-effects models. Binary outcomes, including social media account ownership, secret account ownership, and exposure to bullying and cyberbullying, were analyzed using logistic mixed-effects models. Smartphone acquisition served as the independent variable in all models, and analyses adjusted for the full set of covariates described above.

Mediation analyses were conducted to evaluate whether changes in exposomic factors accounted for a part of the association between smartphone acquisition and subsequent mental health outcomes. Mediation analyses used total BPM score at the 3-year follow-up as the only outcome due to high correlations among BPM subscales (eSupplementary Figure 2). The mediators and total BPM score were assessed at the same follow-up visit. We examined three mediators: total screen time, social media account ownership, and a composite measure of bedtime media use, calculated as the average of video streaming and videogaming before bedtime. Candidate mediators were selected based on the strength of associations observed in mixed-effects models and their conceptual relevance to hypothesized behavioral and environmental pathways.

We fit a single multiple-mediator path model that simultaneously included three candidate mediators. The model included separate exposure-to-mediator pathways for each mediator and a single outcome equation containing smartphone acquisition and all three mediators. Statistical uncertainty for the indirect associations was evaluated using nonparametric bootstrap confidence intervals based on 1,000 bootstrap samples.

All mediation models adjusted for age, sex, race and ethnicity, household income, caregiver education, pubertal status, parental monitoring, and ownership of other personal digital devices. Because social media account ownership and bedtime social media use were highly correlated (eSupplementary Figure 3), bedtime social media use was not included in mediation models to avoid multicollinearity; social media account ownership was retained as the primary indicator of social media engagement. Baseline values of the mediators and BPM were not included in mediation models. Because mediators and outcomes were assessed at the same follow-up time point, mediation results should be interpreted as potential explanatory pathways rather than evidence of temporal ordering.

All statistical analyses were conducted using R statistical software (version 4.5.1; R Foundation for Statistical Computing, Vienna, Austria). Mixed-effects regression models were fitted using the lme4 package, and mediation analyses were performed using the lavaan package. Missing data were handled using complete-case analysis (listwise deletion). Because the dataset contained a single observation per participant, participants with missing values for variables required for a given analysis were excluded from the corresponding analytic sample. The extent of missingness for study variables is reported in Table 1. Analytic sample sizes varied across analyses because descriptive statistics, group comparisons, regression models, and mediation analyses were each conducted using complete cases for the variables included in the corresponding analysis.

**Table 1:** Comparing youth based on smartphone acquisition by year 3 among ABCD Study participants without smartphones at year 2.

Characteristic	Overall	Did not acquire a smartphone	Acquired a smartphone	*P* value	Missing (%)
n	3,486	1,940	1,546		
Age, mean (SD) (year 2 assessment), y	11.82 (0.63)	11.79 (0.62)	11.85 (0.64)	0.004	0.03
Sex female, *n* (%)	1,458 (41.82)	775 (39.95)	683 (44.18)	0.01	0.03
Race and Ethnicity^a^					
White, *n* (%)	2,897 (83.10)	1,640 (84.54)	1,257 (81.31)	0.02	0.2
Black, *n* (%)	421 (12.08)	200 (10.31)	221 (14.29)	<0.001	0.2
Hispanic, *n* (%)	552 (15.83)	276 (14.23)	276 (17.85)	0.004	1.49
Annual household income (year 3 assessment), mean (SD)^b^	7.94 (2.09)	8.00 (2.00)	7.87 (2.19)	0.654	6.63
Caregiver Education: Master's or Higher (year 3 assessment), *n* (%)^c^	1,582 (45.38)	941 (48.51)	641 (41.46)	<0.001	0.2
Pre-smartphone Clinical Characteristics (year 2 assessment)					
Total BPM T Score, mean (SD)^d^	53.31 (5.46)	53.17 (5.38)	53.48 (5.57)	0.069	11.42
BPM Externalizing Symptoms T Score, mean (SD)^d^	52.04 (4.20)	52.06 (4.18)	52.02 (4.22)	0.451	5.22
BPM Attention Symptoms T Score, mean (SD)^d^	55.59 (6.81)	55.34 (6.70)	55.90 (6.93)	0.018	7.40
BPM Internalizing Symptoms T Score, mean (SD)^d^	53.06 (5.18)	53.00 (5.11)	53.14 (5.26)	0.642	4.16
Cyber bullied, *n* (%)	252 (7.23)	121 (6.24)	131 (8.47)	0.014	0.37
Having a social media account, *n* (%)	1,054 (30.24)	469 (24.18)	585 (37.84)	<0.001	0.26
Having a secret social media account, *n* (%)	55 (1.58)	24 (1.24)	31 (2.01)	0.99	70.02
School absenteeism, mean (SD)	0.05 (0.35)	0.05 (0.33)	0.06 (0.37)	0.497	2.32
School grades, mean (SD)	3.04 (1.95)	3.00 (1.89)	3.09 (2.02)	0.57	3.2
Sleep duration on schooldays, mean (SD)	9.23 (1.13)	9.29 (1.15)	9.16 (1.10)	<0.001	1.26
Total screentime, mean (SD)	22.90 (19.76)	20.84 (18.42)	25.49 (21.05)	<0.001	0.17
Active Days ≥60 min, mean (SD)	3.90 (2.17)	3.91 (2.15)	3.88 (2.20)	0.702	0.17
Bullied, *n* (%)	350 (10.04)	194 (10.00)	156 (10.09)	0.183	0.11
Bedtime streaming, mean (SD)	1.57 (0.94)	1.49 (0.88)	1.67 (0.99)	<0.001	0.37
Bedtime videogaming, mean (SD)	1.33 (0.75)	1.30 (0.71)	1.37 (0.79)	0.008	0.40
Bedtime social media, mean (SD)	1.17 (0.54)	1.11 (0.43)	1.24 (0.64)	<0.001	0.40

Group differences in continuous variables were evaluated using nonparametric tests to reduce reliance on distributional assumptions. Specifically, the Wilcoxon rank-sum test was used for comparisons between two groups, and the Kruskal–Wallis test was used when more than two groups were compared. Differences in categorical variables were assessed using Pearson's chi-square test, with Fisher's exact test used when appropriate. All statistical tests were two-sided, and a *p*-value <0.05 was considered statistically significant.

a Race and ethnicity data were reported by each youth participant's caregiver.

b Income is expressed as the mean of the values from 10 self-reported income brackets: (1) <$5,000; (2) $5,000 to $11,999; (3) $12,000 to $15,999; (4) $16,000 to $24,999; (5) $25,000 to $34,999; (6) $35,000 to $49,999; (7) $50,000 to $74,999; (8) $75,000 to $99,999; (9) $100,000 to $199,999; and (10) over $200,000.

c Parental education represents the highest level of education attained by either parent.

d BPM T-scores are norm-referenced measures of symptom severity, with higher scores indicating greater symptom severity.

## 3 Results

Among 3,486 adolescents, 1,546 (44.3%) acquired a smartphone between ages 12 and 13, whereas 1,940 (55.7%) did not (Table 1). The mean (SD) age at baseline was 11.82 (0.63) years and was slightly higher among those who later acquired a smartphone (11.85 vs 11.79 years). Smartphone acquirers were more likely to be female (44.18% vs 39.95%) and had a higher proportion of Black (14.29% vs 10.31%) and Hispanic (17.85% vs 14.23%) adolescents, and a lower proportion of White adolescents (81.31% vs 84.54%). Household income was similar between groups. Caregivers of smartphone acquirers were less likely to have a master's degree or higher (41.46% vs 48.51%).

### 3.1 Smartphone acquisition and mental health outcomes

We first examined whether smartphone acquisition over the prior year was associated with various mental health symptom domains at the 3-year follow-up. In adjusted models, adolescents who acquired a smartphone exhibited greater levels of internalizing symptoms (*β* = 0.11, 95% CI 0.05–0.18), attention problems (*β* = 0.09, 95% CI 0.03–0.16), and total mental health symptom burden (*β* = 0.10, 95% CI 0.04–0.17) relative to those who did not acquire a smartphone (Figure 2). Although statistically significant, the observed associations were small in magnitude, corresponding to approximately 0.09–0.11 standard deviation differences in mental health outcomes. Smartphone acquisition was not associated with differences in externalizing symptoms (*β* = 0.04, 95% CI −0.03–0.10). Full model results are provided in eSupplementary Table 2.

**Figure 2:**
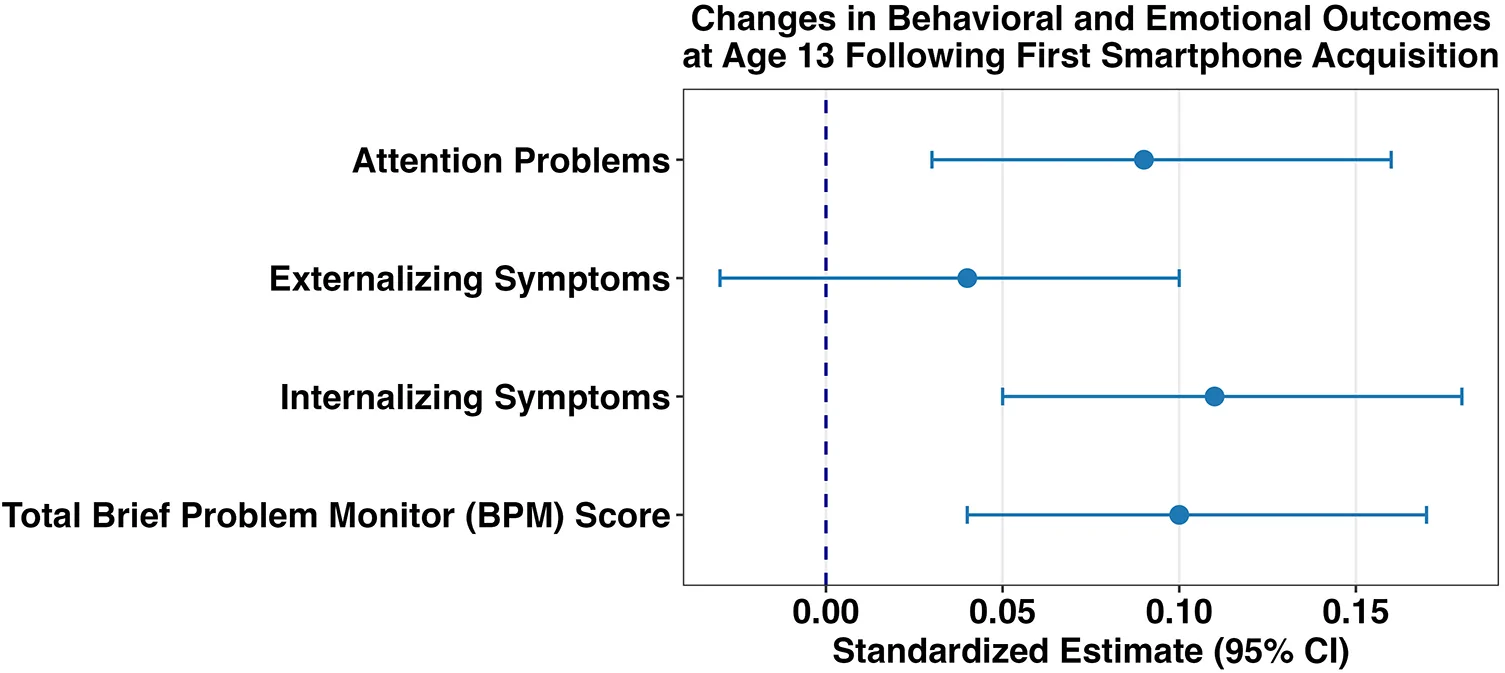
Associations of first smartphone acquisition between ages 12 and 13 with mental health outcomes at age 13. **Legend:** Models were estimated among participants without smartphone ownership at the 2-year assessment in the Adolescent Brain Cognitive Development (ABCD) Study and evaluated associations of first smartphone acquisition between the 2-year and 3-year assessments with mental health outcomes measured at the 3-year assessment. Outcomes were based on the self-reported Brief Problem Monitor (BPM) and included attention problems, externalizing symptoms, internalizing symptoms and total BPM score. Note that for these analyses, T-scores were converted to z-scores so that standardized *β* coefficients reflected the expected scale of standardized effect estimates. All models adjusted for age, sex, race and ethnicity, household income, caregiver education, pubertal status, parental monitoring, ownership of other personal digital devices, and the baseline value of the corresponding outcome measured prior to smartphone acquisition. Estimates are presented as standardized regression coefficients (*β*) with 95% confidence intervals. Points indicate effect estimates and horizontal bars indicate 95% confidence intervals. The vertical dashed line indicates the null value (*β* = 0).

### 3.2 Associations between smartphone acquisition and changes in youth's exposome

#### 3.2.1 Digital exposome measures

Smartphone acquisition was associated with greater screen time duration (*β* = 0.19, 95% CI 0.13–0.25) and increased odds of having a social media account (OR = 3.22, 95% CI 2.66–3.91). No clear association was observed with cyberbullying exposure or secret social media account ownership, although estimates for the latter were imprecise because of the limited sample size available for this analysis (OR = 1.25, 95% CI 0.62–2.51; Figure 3A, eSupplementary Table 3).

**Figure 3:**
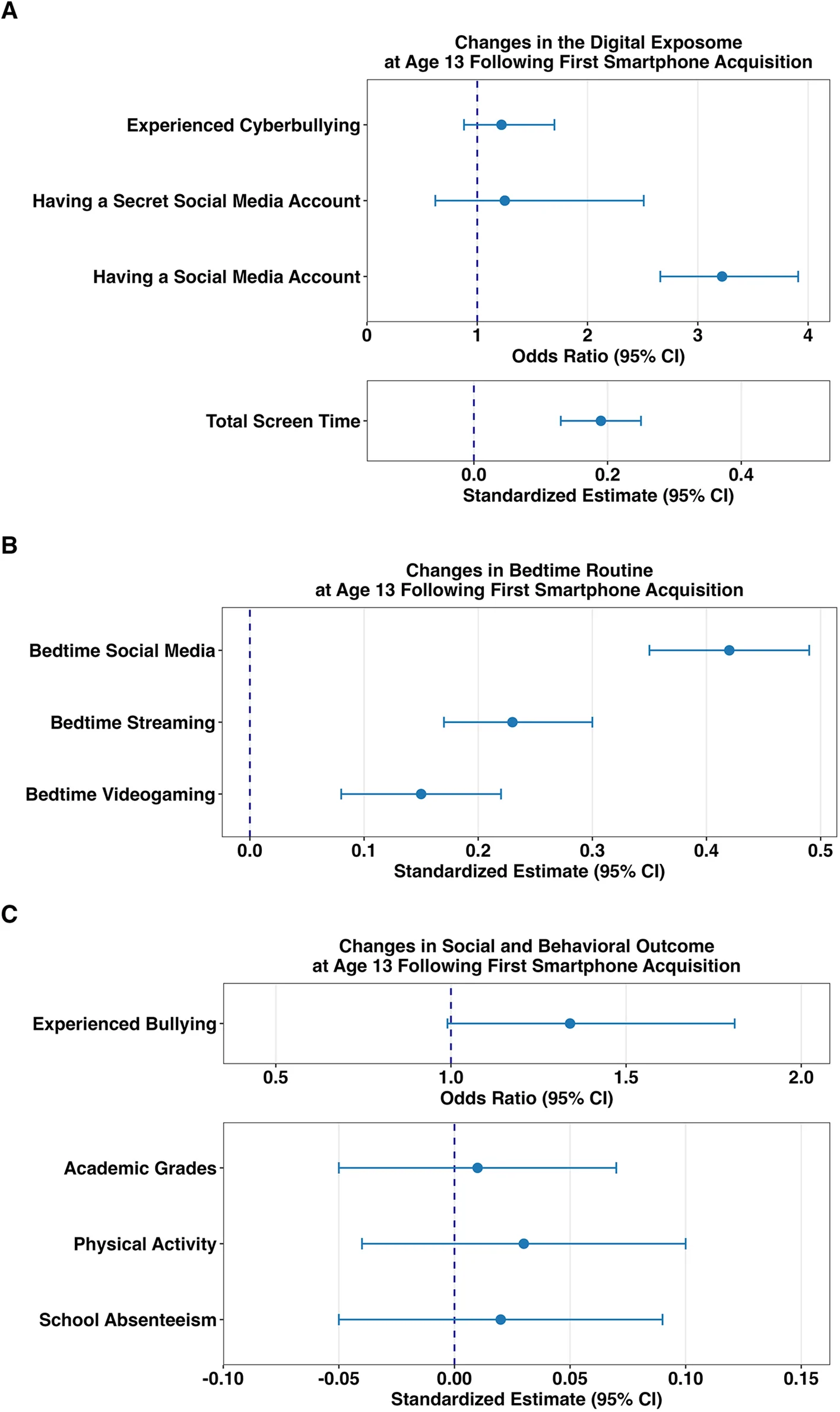
Associations of first smartphone acquisition between ages 12 and 13 with changes in digital, nighttime, and non-digital exposure measures at age 13. **Legend:** Models were estimated among participants without smartphone ownership at the 2-year assessment in the Adolescent Brain Cognitive Development (ABCD) Study and evaluated associations of first smartphone acquisition between the 2-year and 3-year assessments with exposure measures assessed at the 3-year follow-up. Panel **A** shows digital exposure measures (social media account ownership, secret social media account ownership, exposure to cyberbullying, and total screen time). Panel **B** shows bedtime media use (social media use, video streaming, and video gaming). Panel **C** shows non-digital exposures (bullying victimization, academic performance, physical activity, and school absenteeism). All models adjusted for age, sex, race and ethnicity, household income, caregiver education, pubertal status, parental monitoring, ownership of other personal digital devices, and baseline values of the corresponding outcome measured prior to smartphone acquisition. Binary outcomes are presented as odds ratios (OR), and continuous outcomes as standardized regression coefficients (*β*), each with 95% confidence intervals. Points indicate effect estimates and horizontal bars indicate 95% confidence intervals. Vertical dashed lines indicate the null value (OR = 1 for binary outcomes; *β* = 0 for continuous outcomes).

#### 3.2.2 Bedtime routines

Smartphone acquisition was associated with more frequent engagement in bedtime screen use across modalities, including social media (*β* = 0.42, 95% CI 0.35–0.49), video streaming (*β* = 0.23, 95% CI 0.17–0.30), and video gaming (*β* = 0.15, 95% CI 0.08–0.22, Figure 3B, eSupplementary Table 4). Consistent with these changes, smartphone acquisition was associated with shorter sleep duration on school days (*β* = −0.18, 95% CI −0.25 – −0.11). Bedtime reading was inversely associated with smartphone ownership (*β* = −0.27, 95% CI −0.32 - −0.23), note that this specific model was based on cross-sectional data and did not adjust for pre-exposure reading as this data was only available at age 12.

#### 3.2.3 Offline exposome measures

Smartphone acquisition did not show statistically significant associations with changes in offline exposome measures including academic performance, school absenteeism, physical activity, or offline bullying (all p’s > 0.05; Figure 3C, eSupplementary Table 5).

### 3.3 Mediation analyses

We next examined potential indirect associations linking smartphone acquisition, exposomic factors, and mental health symptoms in the subsequent year. The estimates were obtained from a single multiple-mediator model in which (i) total screen time, (ii) social media account ownership, and (iii) bedtime media use (streaming or video-gaming) were included simultaneously. Smartphone acquisition was indirectly associated with mental health symptoms through all three mediators. The standardized indirect association was 0.018 through total screen time (*p* < 0.001), 0.023 through social media account ownership (*p* < 0.001), and 0.011 through bedtime media use (*p* = 0.002). The total standardized indirect association was approximately 0.052, compared with a total standardized association of approximately 0.065. Thus, the three indirect associations collectively represented approximately 80% of the total observed association. Among the variables examined, social media account ownership showed the largest indirect association (35.54%), followed by total screen time (27.86%) and bedtime media use (16.89%) (Figure 4, eSupplementary Table 6). Although the indirect associations represented a substantial proportion of the total observed association, the overall association between smartphone acquisition and mental health symptoms was modest in magnitude. Accordingly, these findings should be interpreted as identifying potentially relevant behavioral pathways rather than large clinically meaningful effects at the individual level.

**Figure 4:**
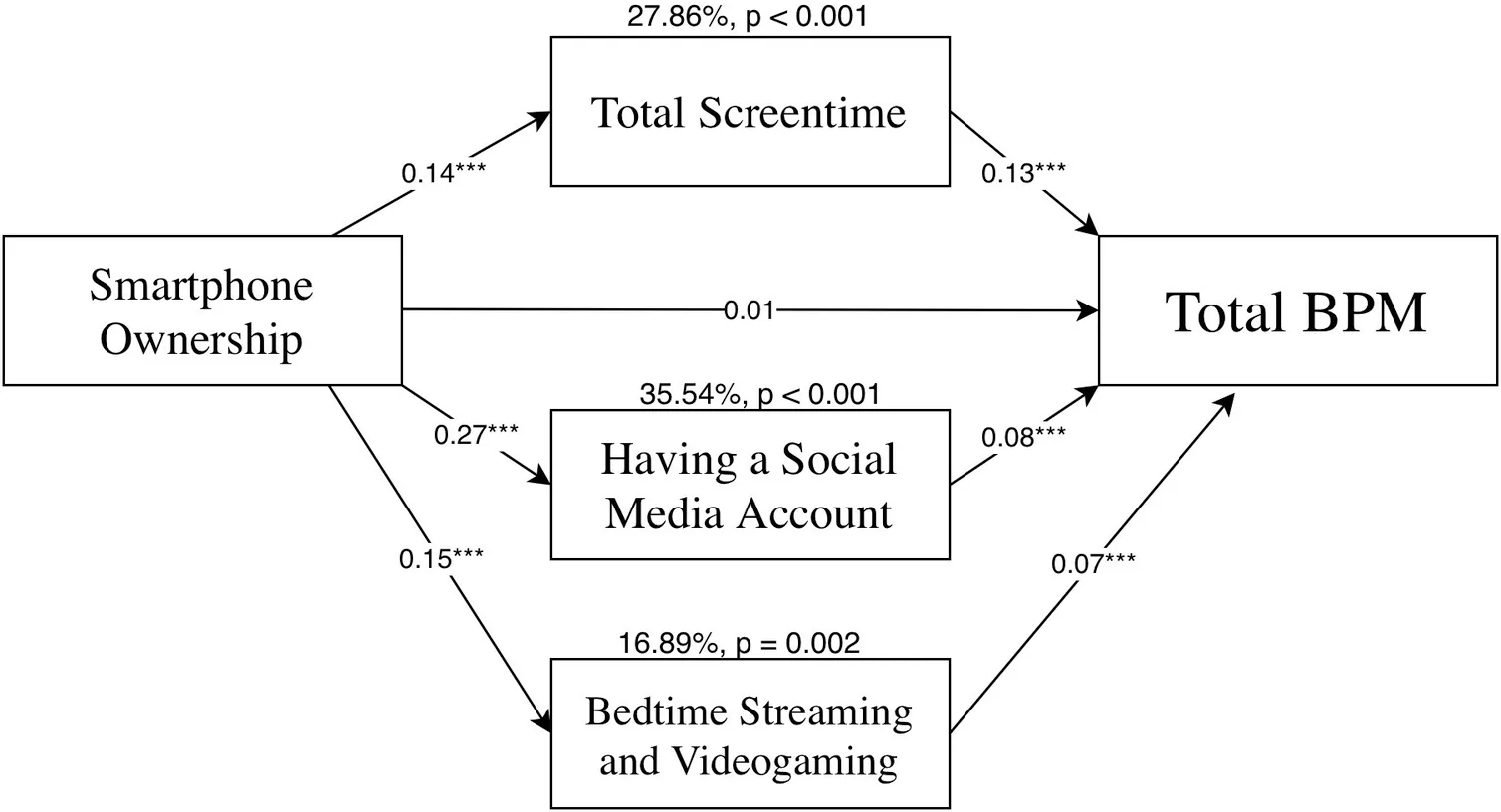
Mediation analysis of associations between first smartphone acquisition and total mental health symptoms through digital exposure measures at age 13. **Legend:** Mediating pathways underlying the association between smartphone acquisition (between the 2-year and 3-year assessments) and subsequent mental health symptoms, measured using the Brief Problem Monitor (BPM) Total Problems score. Three potential mediators were examined: total screen time, social media account ownership, and bedtime streaming and video gaming. Path coefficients represent standardized regression estimates. Percentages shown along each indirect pathway indicate the proportion of the total association explained by that specific mediator, along with the corresponding *p*-value for the indirect effect. For visual clarity, the figure presents a simplified representation of the mediation model. Because mediators and outcomes were assessed at the same follow-up timepoint, the temporal sequence between mediators and mental health symptoms cannot be definitively established. All models were adjusted for age, sex, race and ethnicity, household income, caregiver education, pubertal status, parental monitoring, and ownership of other personal digital devices.

### 3.4 Sensitivity analysis

We repeated the primary analyses using the dichotomized versions of the continuous outcomes. Results from these sensitivity analyses were consistent in direction and statistical significance with those from the primary continuous-outcome models and are presented in the Supplementary Materials (eSupplementary Figures 4–7).

## 4 Discussion

In this longitudinal analysis of adolescents initially without smartphones at age 12, first smartphone acquisition by age 13 was associated with several changes across the adolescent exposome, particularly within digital and sleep-related domains. These changes included greater total screen time, a ∼3-fold greater likelihood of social media account ownership, increased engagement in bedtime media use, and shorter sleep duration. In contrast, offline domains including academic performance, school attendance, physical activity, and bullying, did not show significant evidence of change following smartphone acquisition over the same period. Against this backdrop of exposomic change, smartphone acquisition was also associated with more mental health problems, specifically internalizing and attention symptoms, but not externalizing symptoms. Mediation analyses indicated that shifts in digital exposures, particularly social media account ownership, total screen time, and bedtime media use, accounted for a substantial proportion of the association between smartphone acquisition and mental health outcomes, supporting a model in which changes in the adolescent exposome partially explain observed differences in mental health from pre to post smartphone acquisition.

These findings align with a growing body of literature suggesting that early adolescence is a sensitive period during which digital exposures may have measurable associations with poorer mental health (27, 35). Prior studies have linked both smartphone ownership and screen-based behaviors to internalizing symptoms, sleep disruption, and attentional difficulties (5–7, 36). The present study extends this body of evidence by isolating smartphone acquisition as a developmental transition rather than focusing exclusively on patterns of use among already-exposed youth. By comparing adolescents who acquired smartphones with those who remained without them over the same interval, and adjusting for pre-smartphone exposure measures, this study provides rigorous evidence that the transition into smartphone ownership itself may represent a meaningful shift in risk exposure. The results also lend empirical support to the ‘digital exposome’ framework: Smartphone acquisition appears to act as an entry point into a broader constellation of interconnected exposures, including increased access to social media, greater overall screen engagement, and changes in the temporal structure of daily routines, particularly at night; these findings are in concordance with prior work showing that smartphone adoption coincides with clustered digital behaviors and broader shifts in media use, including more internet and smartphone-based communication (27, 47, 48). Rather than functioning as a single, isolated exposure, the acquisition of the first smartphone appears to reshape multiple dimensions of the adolescent environment simultaneously. This is evident in the strong associations observed for both daytime (overall screen time) and nighttime (bedtime media use, reduced sleep duration) behaviors.

Notably, the strongest behavioral change that we observed following smartphone acquisition was the transition into social media engagement, as reflected by more than threefold increase in the odds of having a social media account among smartphone acquirers. Mediation analyses further indicated that social media account ownership accounted for the largest proportion of the association with mental health outcomes. This pattern is consistent with the central role of social media in peer interaction and social evaluation during adolescence. Increased access to these platforms may amplify opportunities for social comparison, exposure to feedback, and emotional reactivity, potentially contributing to internalizing symptoms (12, 49).

Sleep disruption emerged as another prominent pathway. Adolescents who acquired smartphones reported greater engagement in bedtime media use across multiple modalities and shorter sleep duration. These findings are consistent with prior work linking evening device use to delayed sleep onset and reduced sleep quantity, as well as evidence highlighting the role of cognitive and emotional arousal and light exposure in shaping sleep timing (16, 50). The displacement of bedtime reading observed in cross-sectional analyses further supports the interpretation that smartphone acquisition may shift pre-sleep routines toward more stimulating activities.

Despite concerns grounded in the displacement hypothesis, that smartphone use may crowd out physical activity, academic engagement, or in-person social experiences (51), we did not observe significant changes in these domains over the one-year interval examined. This pattern is consistent with the ‘Goldilocks hypothesis’, which posits that digital engagement is not linearly harmful but instead may follow a different pattern, whereby moderate use is unlikely to displace beneficial offline activities and may even be neutral or adaptive (52). Within this framework, the initial impact of smartphone acquisition in early adolescence may be concentrated primarily within digital contexts and closely related behaviors, rather than broadly altering offline functioning. It is also possible that shifts in physical, academic, or social activities emerge only at higher levels of use or over longer developmental periods, when engagement moves beyond this moderate range.

Our findings may have public health implications. First, they underscore the importance of conceptualizing smartphone acquisition as a pivotal developmental milestone. The transition into personal device ownership introduces a qualitatively new exposure context, characterized by continuous, private, and largely unsupervised access to digital environments, which may shape trajectories of behavior and mental health. Second, as smartphone acquisition may represent a useful window for anticipatory guidance and prevention, the transition to smartphone ownership provides an opportunity for clinicians, caregivers, and schools to promote healthy digital habits, particularly around social media engagement, screen time, and bedtime device use, before these behaviors become established. The identification of modifiable behavioral pathways suggests potential targets for intervention. The mediating role of social media engagement, screen time, and bedtime media use indicates that risk is not inherent to smartphone ownership *per se*, but may arise through specific patterns of use. Interventions aimed at promoting healthier digital habits—such as limiting nighttime device use, encouraging structured boundaries around screen time, and supporting mindful engagement with social media—may help mitigate potential negative outcomes. Third, the study contributes to a broader shift toward integrative models of digital exposures, emphasizing that multiple, interrelated behaviors, rather than single metrics such as total screen time, may provide a more informative understanding of risk. Importantly, these findings highlight the need to more explicitly position smartphone acquisition as a key health-related exposure, akin to established behavioral factors such as physical activity and nutrition, with important implications for future research. Advancing this area will require greater attention to the timing and context of device uptake, as well as increased use of objective, high-resolution tracking data to more precisely capture patterns of use and their links to developmental outcomes.

Several limitations warrant consideration. First, although the longitudinal design and adjustment for baseline measures strengthen temporal inference, causal conclusions cannot be definitively established. Residual confounding may persist, including unmeasured factors related to family context, parenting practices, or adolescent characteristics that influence both smartphone acquisition and outcomes. Second, measures of digital behavior were based on self-report and caregiver report. Reliance on a common measurement source may introduce shared-source bias, recall bias or misclassification, whereby correlated measurement error or response tendencies may influence observed associations. This limitation may be particularly relevant to the mediation analyses, and future studies should incorporate objective measures and multiple informants where feasible. Third, the observational window was limited to a single one-year interval following smartphone acquisition. This relatively short timeframe may capture only the initial adjustment period and may not reflect longer-term trajectories or potential adaptation effects. Fourth, the operationalization of smartphone acquisition does not capture heterogeneity in device use practices following acquisition. Adolescents may differ widely in how they engage with their smartphones, the types of platforms they use, and the degree of parental monitoring, all of which may moderate observed associations. Fifth, although the ABCD Study provides a large and diverse sample, findings may not generalize to populations with different cultural, technological, or policy contexts, particularly given rapid changes in digital platforms and norms. Moreover, because smartphone acquisition was examined only between ages 12 and 13, the extent to which these findings generalize to acquisition at younger ages remains unknown. Sixth, mediation analyses should be interpreted cautiously. Although smartphone acquisition preceded assessment of the mediators and mental health outcomes, the mediators and BPM outcomes were measured at the same follow-up assessment. As a result, the temporal ordering between changes in digital exposures and mental health symptoms cannot be definitively established, and reverse or bidirectional relationships remain possible. In addition, mediation analyses rely on assumptions regarding unmeasured confounding that cannot be fully verified in observational data. Finally, missing data resulted in substantial reductions in analytic sample sizes for some models, including the primary BPM model. Because analyses were conducted using complete cases, selection bias may have been introduced if missingness was associated with smartphone acquisition, covariates, or outcomes. Missingness was particularly pronounced for the secret social media account variable, resulting in less precise estimates for that analysis. Accordingly, these findings should be interpreted with caution.

In summary, our findings support the conceptualization of smartphone acquisition as an inflection point in the adolescent exposome, marking the transition into a sustained and multifaceted digital environment. Rather than reflecting a single exposure, smartphone ownership appears to initiate coordinated changes across interconnected behavioral domains that are relevant to mental health. The observation that these changes were statistically associated with a substantial proportion of the observed relationship between smartphone acquisition and mental health outcomes highlights the importance of considering mechanisms linking digital environments to adolescent well-being, rather than focusing on device ownership alone. Future research should extend these findings by examining longer-term developmental trajectories and earlier developmental timing of acquisition, identifying individual differences in susceptibility, and incorporating objective measures of digital behavior. Experimental and quasi-experimental designs—including interventions targeting patterns of use or variation in the timing of smartphone acquisition—will be critical for strengthening causal inference. From a practical perspective, the period of smartphone introduction may represent a key window for intervention, during which efforts to support healthier digital habits could help mitigate potential adverse effects on adolescent mental health.

## Data Availability

The data analyzed in this study are subject to the following licenses/restrictions: data from the Adolescent Brain Cognitive Development (ABCD) Study (Data Release 5.1) are available to qualified researchers through the NIMH Data Archive (NDA) upon request and approval. Requests to access these datasets should be directed to the NIMH Data Archive, https://nda.nih.gov/.
