# Supplementary material for: Smartphone acquisition as an inflection point in early adolescence digital exposome: relevance to mental health
Source: Front Child Adolesc Psychiatry. 2026 Sep 30;5:1940646. doi: 10.3389/frcha.2026.1940646 (PMC13662111; doi:10.3389/frcha.2026.1940646)
Supplement: Supplementary file 1 [file Datasheet1.docx]

**Supplemental Material**

**Smartphone Acquisition as an Inflection Point in Early Adolescence Digital Exposome: Relevance to Mental Health**

**Table of contents:**

**eSupplementary Table 1 -** Comparison of ABCD Study participants who were included in the analyses (did not have a smartphone before the 2-year assessment) to those who are not included in the analyses (who acquired a smartphone before the 2-year assessment).

**eSupplementary Table 2 -** Associations of first smartphone acquisition between ages 12 and 13 with mental health outcomes at age 13.

**eSupplementary Table 3 -** Associations of first smartphone acquisition between ages 12 and 13 with changes in digital exposure measures at age 13.

**eSupplementary Table 4 -** Associations of first smartphone acquisition between ages 12 and 13 with changes in nighttime exposure measures at age 13.

**eSupplementary Table 5 -** Associations of first smartphone acquisition between ages 12 and 13 with changes in non-digital exposure measures at age 13.

**eSupplementary Table 6 -** Mediation analysis of associations between first smartphone acquisition and total mental health symptoms through digital exposure measures at age 13.

**eSupplementary Figure 1 -** Distribution of Continuous Variables Included in Primary Analyses

**eSupplementary Figure 2 -** Intercorrelations Among Total BPM, Internalizing Symptoms, and Attention T-Scores

**eSupplementary Figure 3 -** Intercorrelations Among Significant Screen Use Variables Considered for Mediation Analysis

**eSupplementary Figure 4 -** Sensitivity Analysis Using Dichotomized Mental Health Outcomes

**eSupplementary Figure 5 -** Sensitivity Analysis Using Dichotomized Digital Exposome Outcomes

**eSupplementary Figure 6 -** Sensitivity Analysis Using Dichotomized Bedtime Routine Outcomes

**eSupplementary Figure 7 -** Sensitivity Analysis Using Dichotomized Social and Behavioral Outcomes

**eSupplementary Table 1 -** Comparison of ABCD Study participants who were included in the analyses (did not have a smartphone before the 2-year assessment) to those who are not included in the analyses (who acquired a smartphone before the 2-year assessment).

|  | **Overall** | **No Smartphone** | **Yes Smartphone** | **P value** | **Missing (%)** |  |
| --- | --- | --- | --- | --- | --- | --- |
| **n** | 10,588 | 3,849 | 6,739 |  |  |  |
| **Age, mean (SD) (year 2 assessment), y** | 12.02 (0.67) | 11.83 (0.63) | 12.13 (0.66) | <0.001 | 0.01 |  |
| **Sex female, n (%)** | 5028 (47.49) | 1623 (42.17) | 3405 (50.53) | <0.001 | 0.03 |  |
| **Race and Ethnicity^a^** |  |  |  |  |  |  |
| **White, n (%)** | 7985 (75.42) | 3161 (82.13) | 4824 (71.58) | <0.001 | 0.17 |  |
| **Black, n (%)** | 2153 (20.33) | 492 (12.78) | 1661 (24.65) | <0.001 | 0.17 |  |
| **Hispanic, n (%)** | 2067 (19.52) | 621 (16.13) | 1446 (21.46) | <0.001 | 1.17 |  |
| **Annual household income (year 2 assessment), mean (SD)^b^** | 7.54 (2.31) | 7.86 (2.11) | 7.35 (2.40) | <0.001 | 8.03 |  |
| **Caregiver Education: Master’s or Higher (year 2 assessment), n (%)^c^** | 3834 (36.21) | 1708 (44.38) | 2126 (31.55) | <0.001 | 0.19 |  |
| **Pre-smartphone Clinical Characteristics** | | | | | | |
| **Total BPM T Score, mean (SD)** | 53.71 (5.64) | 53.35 (5.46) | 53.91 (5.73) | <0.001 | 11.46 |  |
| **BPM Externalizing Symptoms T Score, mean (SD)** ^d^ | 52.10 (4.27) | 52.08 (4.24) | 52.11 (4.29) | 0.66 | 5.14 |  |
| **BPM Attention Symptoms T Score, mean (SD)** ^d^ | 56.21 (6.95) | 55.68 (6.84) | 56.51 (6.99) | <0.001 | 7.62 |  |
| **BPM Internalizing Symptoms T Score, mean (SD)** ^d^ | 53.41 (5.41) | 53.09 (5.18) | 53.59 (5.52) | <0.001 | 4.18 |  |
| **Cyber bullied, n (%)** | 951 (8.98) | 281 (7.30) | 670 ( 9.94) | <0.001 | 0.49 |  |
| **Having a social media account, n (%)** | 5830 (55.06) | 1171 (30.42) | 4659 (69.13) | <0.001 | 0.27 |  |
| **Having a secret social media account, n (%)** | 268 ( 2.53) | 63 ( 1.64) | 205 ( 3.04) | 0.17 | 45.26 |  |
| **School absenteeism, mean (SD)** | 0.10 (0.49) | 0.06 (0.36) | 0.12 (0.55) | <0.001 | 2.07 |  |
| **School grades, mean (SD)** | 3.27 (2.06) | 3.07 (1.96) | 3.38 (2.10) | <0.001 | 2.57 |  |
| **Sleep duration on schooldays, mean (SD)** | 8.91 (1.26) | 9.23 (1.13) | 8.73 (1.29) | <0.001 | 1.44 |  |
| **Total screentime, mean (SD)** | 28.58 (22.80) | 23.34 (20.29) | 31.57 (23.60) | <0.001 | 0.23 |  |
| **Active Days ≥60 min, mean (SD)** | 3.77 (2.16) | 3.88 (2.18) | 3.72 (2.14) | <0.001 | 0.22 |  |
| **Bullied, n (%)** | 1105 (10.50) | 384 (10.00) | 721 (10.70) | 0.256 | 0.18 |  |
| **Bedtime streaming, mean (SD)** | 1.85 (1.05) | 1.58 (0.94) | 2.01 (1.08) | <0.001 | 0.4 |  |
| **Bedtime videogaming, mean (SD)** | 1.45 (0.84) | 1.34 (0.76) | 1.51 (0.88) | <0.001 | 0.37 |  |
| **Bedtime social media, mean (SD)** | 1.49 (0.88) | 1.17 (0.55) | 1.67 (0.97) | <0.001 | 0.43 |  |

Group differences in continuous variables were evaluated using nonparametric tests to reduce reliance on distributional assumptions. Specifically, the Wilcoxon rank-sum test was used for comparisons between two groups, and the Kruskal–Wallis test was used when more than two groups were compared. Differences in categorical variables were assessed using Pearson’s chi-square test, with Fisher’s exact test used when appropriate. All statistical tests were two-sided, and a p-value <0.05 was considered statistically significant.

^a^Race and ethnicity data were reported by each youth participant’s caregiver.
^b^Income is expressed as the mean of the values from 10 self-reported income brackets: (1) <$5000; (2) $5000 to 11,999; (3) $12,000 to 15,999; (4) $16,000 to 24,999; (5) $25,000 to 34,999; (6) $35,000 to 49,999; (7) $50,000 to 74,999; (8) $75,000 to 99,999; (9) $100,000 to 199,999; and (10) over $200,000.
^c^Parental education represents the highest level of education attained by either parent.

^d^BPM T-scores are norm-referenced measures of symptom severity, with higher scores indicating greater symptom severity.

**eSupplementary Table 2 -** Associations of first smartphone acquisition between ages 12 and 13 with mental health outcomes at age 13.

|  | **BPM Attention Problems (N = 2,648)** | | | | **BPM Externalizing Symptoms (N=2,745)** | | | | **BPM Internalizing Symptoms (N=2,802)** | | | | **Total BPM Score**  **(N = 2,491)** | | |
| --- | --- | --- | --- | --- | --- | --- | --- | --- | --- | --- | --- | --- | --- | --- | --- |
| **Variable** | *Beta* | *95% CI* | *p* | *Beta* | | *95% CI* | *p* | *Beta* | | *95% CI* | *p* | *Beta* | | *95% CI* | *p* |
| **Past-year smartphone acquisition** | 0.09 | 0.03 – 0.16 | 0.003 | 0.04 | | -0.03 – 0.10 | 0.264 | 0.11 | | 0.05 – 0.18 | **0.001** | 0.1 | | 0.04 – 0.17 | **0.003** |
| **Age^a^** | 0.03 | -0.02 – 0.08 | 0.222 | 0.04 | | -0.01 – 0.09 | 0.138 | 0.02 | | -0.03 – 0.08 | 0.424 | 0.03 | | -0.03 – 0.08 | 0.305 |
| **Sex, female** | 0.18 | 0.11 – 0.26 | <0.001 | 0.07 | | -0.00 – 0.15 | 0.058 | 0.07 | | -0.01 – 0.15 | 0.069 | 0.14 | | 0.07 – 0.22 | **<0.001** |
| **Race, Black^b^** | -0.01 | -0.12 – 0.09 | 0.814 | 0.03 | | -0.08 – 0.14 | 0.571 | -0.03 | | -0.14 – 0.09 | 0.621 | -0.01 | | -0.13 – 0.10 | 0.816 |
| **Ethnicity, Hispanic^b^** | 0.05 | -0.04 – 0.14 | 0.318 | 0 | | -0.10 – 0.09 | 0.961 | 0.05 | | -0.05 – 0.15 | 0.335 | 0.03 | | -0.07 – 0.12 | 0.591 |
| **Household income^a,c^** | -0.01 | -0.03 – 0.01 | 0.459 | 0 | | -0.02 – 0.02 | 0.773 | 0 | | -0.02 – 0.02 | 0.77 | 0 | | -0.02 – 0.02 | 0.884 |
| **Parent education^a,d^** | 0 | -0.03 – 0.02 | 0.665 | 0 | | -0.03 – 0.02 | 0.669 | 0.01 | | -0.01 – 0.03 | 0.42 | 0 | | -0.03 – 0.02 | 0.867 |
| **Pubertal stage^e^** | 0 | -0.04 – 0.04 | 0.875 | 0.04 | | 0.00 – 0.08 | **0.04** | 0.04 | | 0.00 – 0.09 | **0.035** | 0.04 | | -0.00 – 0.08 | 0.075 |
| **Parental monitoring^a^** | -0.23 | -0.29 – -0.16 | <0.001 | -0.27 | | -0.34 – -0.20 | **<0.001** | -0.19 | | -0.26 – -0.12 | **<0.001** | -0.28 | | -0.35 – -0.21 | **<0.001** |
| **Ownership of other devices^a^** | | | | | | | | | | | | | | | |
| **Tablet** | 0.07 | 0.01 – 0.13 | 0.022 | 0.04 | | -0.02 – 0.10 | 0.214 | 0.05 | | -0.02 – 0.11 | 0.162 | 0.06 | | -0.01 – 0.12 | 0.088 |
| **Laptop** | 0.06 | 0.00 – 0.12 | 0.045 | 0.03 | | -0.04 – 0.09 | 0.417 | 0.05 | | -0.02 – 0.11 | 0.162 | 0.07 | | 0.00 – 0.13 | **0.048** |
| **iPod** | 0.07 | -0.02 – 0.16 | 0.145 | -0.02 | | -0.11 – 0.08 | 0.69 | 0.02 | | -0.08 – 0.12 | 0.638 | 0.03 | | -0.06 – 0.13 | 0.501 |
| **Smart watch** | -0.11 | -0.25 – 0.02 | 0.099 | -0.05 | | -0.20 – 0.09 | 0.452 | -0.08 | | -0.22 – 0.07 | 0.298 | -0.2 | | -0.34 – -0.05 | **0.007** |
| **Pre-smartphone health measure** | 0.57 | 0.54 – 0.61 | <0.001 | 0.48 | | 0.44 – 0.51 | **<0.001** | 0.49 | | 0.46 – 0.52 | **<0.001** | 0.52 | | 0.49 – 0.55 | **<0.001** |

^a^The variables age, household income, parent education, parental monitoring, ownership of other devices are from year 3 follow-up
^b^Race and ethnicity data were reported by each youth participant’s caregiver.
^c^Income is expressed as the mean of the values from 10 self-reported income brackets: (1) <$5000; (2) $5000 to 11,999; (3) $12,000 to 15,999; (4) $16,000 to 24,999; (5) $25,000 to 34,999; (6) $35,000 to 49,999; (7) $50,000 to 74,999; (8) $75,000 to 99,999; (9) $100,000 to 199,999; and (10) over $200,000.
^d^Parental education represents the highest level of education attained by either parent.
^e^The variable pubertal stage is from the year 2 follow-up.

**eSupplementary Table 3-** Associations of first smartphone acquisition between ages 12 and 13 with changes in digital exposure measures at age 13.

|  | **Experienced Cyberbullying (N=3,033)** | | | | **Having a Secret Social Media Account (N=759)** | | | | **Having a Social Media Account (N=3,039)** | | | | **Total Screentime**  **(N=3,042)** | | |
| --- | --- | --- | --- | --- | --- | --- | --- | --- | --- | --- | --- | --- | --- | --- | --- |
| **Variable** | *Odds Ratio* | *95% CI* | *p* | *Odds Ratio* | | *95% CI* | *p* | *Odds Ratio* | | *95% CI* | *p* | *Beta* | | *95% CI* | *p* |
| **Past-year smartphone acquisition** | 1.22 | 0.88 – 1.70 | 0.224 | 1.25 | | 0.62 – 2.51 | 0.534 | 3.22 | | 2.66 – 3.91 | <0.001 | 0.19 | | 0.13 – 0.25 | <0.001 |
| **Age^a^** | 0.92 | 0.70 – 1.20 | 0.534 | 1.26 | | 0.74 – 2.15 | 0.386 | 1.1 | | 0.94 – 1.28 | 0.245 | 0 | | -0.05 – 0.05 | 0.879 |
| **Sex, female** | 1.04 | 0.71 – 1.52 | 0.851 | 1.21 | | 0.55 – 2.69 | 0.634 | 0.9 | | 0.72 – 1.12 | 0.344 | -0.06 | | -0.13 – 0.01 | 0.12 |
| **Race, Black^b^** | 1.11 | 0.67 – 1.86 | 0.686 | 1.52 | | 0.66 – 3.50 | 0.328 | 1.57 | | 1.13 – 2.17 | 0.007 | 0.17 | | 0.06 – 0.28 | 0.002 |
| **Ethnicity, Hispanic^b^** | 1.19 | 0.76 – 1.86 | 0.439 | 0.54 | | 0.19 – 1.50 | 0.236 | 1.57 | | 1.19 – 2.07 | 0.001 | 0.06 | | -0.03 – 0.15 | 0.191 |
| **Household income^a,c^** | 1.03 | 0.93 – 1.14 | 0.543 | 0.98 | | 0.82 – 1.17 | 0.787 | 0.97 | | 0.91 – 1.03 | 0.34 | 0 | | -0.02 – 0.01 | 0.64 |
| **Parent education^a,d^** | 0.99 | 0.89 – 1.11 | 0.92 | 0.89 | | 0.72 – 1.10 | 0.289 | 0.97 | | 0.91 – 1.04 | 0.355 | -0.04 | | -0.06 – -0.02 | <0.001 |
| **Pubertal stage^e^** | 1.15 | 0.93 – 1.42 | 0.194 | 1.2 | | 0.77 – 1.89 | 0.422 | 1.26 | | 1.12 – 1.42 | <0.001 | 0.05 | | 0.01 – 0.09 | 0.008 |
| **Parental monitoring^a^** | 0.69 | 0.51 – 0.94 | 0.017 | 0.49 | | 0.27 – 0.90 | 0.02 | 0.64 | | 0.53 – 0.78 | <0.001 | -0.2 | | -0.26 – -0.14 | <0.001 |
| **Ownership of other devices^a^** | | | | | | | | | | | | | | | |
| **Tablet** | 1.25 | 0.91 – 1.72 | 0.172 | 2.34 | | 1.21 – 4.54 | 0.012 | 1.21 | | 1.00 – 1.46 | 0.045 | 0.13 | | 0.07 – 0.19 | <0.001 |
| **Laptop** | 1.25 | 0.91 – 1.73 | 0.171 | 1.18 | | 0.61 – 2.27 | 0.631 | 0.92 | | 0.76 – 1.12 | 0.412 | 0.04 | | -0.02 – 0.10 | 0.194 |
| **iPod** | 0.87 | 0.51 – 1.49 | 0.613 | 1.5 | | 0.59 – 3.77 | 0.392 | 2.34 | | 1.75 – 3.11 | <0.001 | 0 | | -0.09 – 0.09 | 0.984 |
| **Smart watch** | 0.82 | 0.37 – 1.81 | 0.63 | 2.45 | | 0.84 – 7.15 | 0.101 | 1.2 | | 0.78 – 1.84 | 0.401 | -0.04 | | -0.18 – 0.09 | 0.541 |
| **Pre-smartphone health measure** | 10.13 | 7.04 – 14.57 | <0.001 | 7.89 | | 3.36 – 18.53 | <0.001 | 10.61 | | 8.51 – 13.23 | <0.001 | 0.42 | | 0.39 – 0.46 | <0.001 |

^a^The variables age, household income, parent education, parental monitoring, ownership of other devices are from year 3 follow-up
^b^Race and ethnicity data were reported by each youth participant’s caregiver.
^c^Income is expressed as the mean of the values from 10 self-reported income brackets: (1) <$5000; (2) $5000 to 11,999; (3) $12,000 to 15,999; (4) $16,000 to 24,999; (5) $25,000 to 34,999; (6) $35,000 to 49,999; (7) $50,000 to 74,999; (8) $75,000 to 99,999; (9) $100,000 to 199,999; and (10) over $200,000.
^d^Parental education represents the highest level of education attained by either parent.
^e^The variable pubertal stage is from the year 2 follow-up.

**eSupplementary Table 4-** Associations of first smartphone acquisition between ages 12 and 13 with changes in bedtime routine measures at age 13.

|  | **Bedtime Social Media (N=3,032)** | | | | **Bedtime Streaming (N=3,032)** | | | | **Bedtime Videogaming (N=3,034)** | | | | **Bedtime Reading (N=9,091)** | | |
| --- | --- | --- | --- | --- | --- | --- | --- | --- | --- | --- | --- | --- | --- | --- | --- |
| **Variable** | *Beta* | *95% CI* | *p* | *Beta* | | *95% CI* | *p* | *Beta* | | *95% CI* | *p* | *Beta* | | *95% CI* | *p* |
| **Past-year smartphone acquisition** | 0.42 | 0.35 – 0.49 | <0.001 | 0.23 | | 0.17 – 0.30 | <0.001 | 0.15 | | 0.08 – 0.22 | <0.001 | -0.27 | | -0.32 – -0.23 | <0.001 |
| **Age^a^** | 0.05 | -0.01 – 0.10 | 0.08 | -0.01 | | -0.06 – 0.05 | 0.838 | 0.01 | | -0.04 – 0.07 | 0.615 | -0.11 | | -0.14 – -0.08 | <0.001 |
| **Sex, female** | 0.08 | 0.00 – 0.16 | 0.044 | 0.01 | | -0.07 – 0.09 | 0.784 | -0.22 | | -0.30 – -0.14 | <0.001 | 0.17 | | 0.13 – 0.22 | <0.001 |
| **Race, Black^b^** | 0.21 | 0.10 – 0.33 | <0.001 | 0.29 | | 0.17 – 0.41 | <0.001 | 0.19 | | 0.07 – 0.31 | 0.002 | -0.02 | | -0.08 – 0.04 | 0.445 |
| **Ethnicity, Hispanic^b^** | 0.1 | -0.00 – 0.20 | 0.061 | 0.01 | | -0.09 – 0.11 | 0.858 | 0.08 | | -0.02 – 0.18 | 0.129 | -0.17 | | -0.23 – -0.11 | <0.001 |
| **Household income^a,c^** | -0.01 | -0.04 – 0.01 | 0.168 | -0.02 | | -0.04 – 0.01 | 0.151 | -0.03 | | -0.05 – -0.01 | 0.004 | 0.01 | | -0.01 – 0.02 | 0.286 |
| **Parent education^a,d^** | -0.03 | -0.06 – -0.01 | 0.004 | -0.05 | | -0.08 – -0.03 | <0.001 | -0.04 | | -0.06 – -0.02 | 0.001 | 0.07 | | 0.05 – 0.08 | <0.001 |
| **Pubertal stage^e^** | 0.05 | 0.01 – 0.09 | 0.024 | 0.02 | | -0.02 – 0.06 | 0.422 | 0.02 | | -0.02 – 0.06 | 0.374 | 0 | | -0.03 – 0.02 | 0.762 |
| **Parental monitoring^a^** | -0.13 | -0.19 – -0.06 | <0.001 | -0.09 | | -0.16 – -0.02 | 0.011 | -0.16 | | -0.23 – -0.09 | <0.001 | 0.21 | | 0.17 – 0.26 | <0.001 |
| **Ownership of other devices^a^** | | | | | | | | | | | | | | | |
| **Tablet** | 0.03 | -0.04 – 0.09 | 0.399 | 0.1 | | 0.03 – 0.16 | 0.004 | 0.1 | | 0.03 – 0.16 | 0.006 | -0.02 | | -0.06 – 0.02 | 0.231 |
| **Laptop** | 0 | -0.07 – 0.07 | 0.997 | 0.06 | | -0.01 – 0.13 | 0.072 | -0.04 | | -0.11 – 0.03 | 0.291 | 0 | | -0.04 – 0.05 | 0.894 |
| **iPod** | 0.2 | 0.10 – 0.31 | <0.001 | 0.07 | | -0.03 – 0.17 | 0.198 | 0.05 | | -0.05 – 0.16 | 0.323 | -0.03 | | -0.10 – 0.03 | 0.346 |
| **Smart watch** | 0.09 | -0.06 – 0.24 | 0.23 | -0.07 | | -0.22 – 0.08 | 0.35 | -0.06 | | -0.21 – 0.10 | 0.477 | -0.09 | | -0.17 – -0.00 | 0.046 |
| **Pre-smartphone health measure** | 0.29 | 0.26 – 0.33 | <0.001 | 0.27 | | 0.24 – 0.31 | <0.001 | 0.2 | | 0.16 – 0.24 | <0.001 | NA | | | |

^a^The variables age, household income, parent education, parental monitoring, ownership of other devices are from year 3 follow-up
^b^Race and ethnicity data were reported by each youth participant’s caregiver.
^c^Income is expressed as the mean of the values from 10 self-reported income brackets: (1) <$5000; (2) $5000 to 11,999; (3) $12,000 to 15,999; (4) $16,000 to 24,999; (5) $25,000 to 34,999; (6) $35,000 to 49,999; (7) $50,000 to 74,999; (8) $75,000 to 99,999; (9) $100,000 to 199,999; and (10) over $200,000.
^d^Parental education represents the highest level of education attained by either parent.

^e^The variable pubertal stage is from the year 2 follow-up.

**eSupplementary Table 5-** Associations of first smartphone acquisition between ages 12 and 13 with changes in non-digital exposure measures at age 13.

|  | **Experienced Bullying (N=3,044)** | | | | **Grades**  **(N=2,874)** | | | | **Physical Activity**  **(N=3,041)** | | | | **School Absenteeism**  **(N=2,924)** | | | | **Sleep Duration on School Days (N=3,006)** | | |
| --- | --- | --- | --- | --- | --- | --- | --- | --- | --- | --- | --- | --- | --- | --- | --- | --- | --- | --- | --- |
| **Variable** | *Odds Ratio* | *95% CI* | *p* | *Beta* | | *95% CI* | *p* | *Beta* | | *95% CI* | *p* | *Beta* | | *95% CI* | *p* | *Beta* | | *95% CI* | *p* |
| **Past-year smartphone acquisition** | 1.34 | 0.99 – 1.81 | 0.055 | 0.01 | | -0.05 – 0.07 | 0.723 | 0.03 | | -0.04 – 0.10 | 0.338 | 0.02 | | -0.05 – 0.09 | 0.631 | -0.18 | | -0.25 – -0.11 | <0.001 |
| **Age^a^** | 0.87 | 0.68 – 1.11 | 0.273 | 0.07 | | 0.02 – 0.11 | 0.01 | -0.06 | | -0.11 – 0.00 | 0.052 | 0.04 | | -0.02 – 0.10 | 0.147 | -0.12 | | -0.17 – -0.07 | <0.001 |
| **Sex, female** | 0.79 | 0.55 – 1.13 | 0.194 | -0.09 | | -0.16 – -0.01 | 0.018 | -0.09 | | -0.17 – -0.01 | 0.032 | -0.01 | | -0.09 – 0.08 | 0.865 | 0.04 | | -0.04 – 0.12 | 0.308 |
| **Race, Black^b^** | 1.25 | 0.80 – 1.96 | 0.334 | 0.19 | | 0.09 – 0.30 | <0.001 | -0.11 | | -0.23 – 0.00 | 0.06 | 0.12 | | 0.00 – 0.25 | 0.049 | -0.13 | | -0.25 – -0.02 | 0.022 |
| **Ethnicity, Hispanic^b^** | 0.88 | 0.56 – 1.39 | 0.586 | 0.02 | | -0.07 – 0.11 | 0.715 | -0.12 | | -0.22 – -0.02 | 0.02 | 0.08 | | -0.03 – 0.18 | 0.148 | 0.01 | | -0.09 – 0.10 | 0.913 |
| **Household income^a,c^** | 0.94 | 0.86 – 1.02 | 0.149 | -0.04 | | -0.06 – -0.02 | <0.001 | 0.02 | | -0.00 – 0.04 | 0.08 | -0.04 | | -0.06 – -0.01 | 0.002 | 0 | | -0.02 – 0.02 | 0.757 |
| **Parent education^a,d^** | 1.17 | 1.05 – 1.31 | 0.004 | -0.06 | | -0.08 – -0.03 | <0.001 | 0.02 | | -0.01 – 0.04 | 0.17 | -0.02 | | -0.05 – 0.00 | 0.077 | 0 | | -0.02 – 0.03 | 0.724 |
| **Pubertal stage^e^** | 1.22 | 1.00 – 1.48 | 0.048 | 0.03 | | -0.01 – 0.07 | 0.105 | -0.03 | | -0.07 – 0.01 | 0.155 | 0.04 | | -0.00 – 0.09 | 0.075 | -0.11 | | -0.15 – -0.07 | <0.001 |
| **Parental monitoring^a^** | 0.56 | 0.43 – 0.74 | <0.001 | -0.26 | | -0.32 – -0.20 | <0.001 | 0.17 | | 0.10 – 0.24 | <0.001 | -0.2 | | -0.27 – -0.12 | <0.001 | 0.14 | | 0.07 – 0.20 | <0.001 |
| **Ownership of other devices^a^** | | | | | | | | | | | | | | | | | | | |
| **Tablet** | 0.92 | 0.68 – 1.24 | 0.595 | -0.01 | | -0.07 – 0.05 | 0.671 | 0.02 | | -0.05 – 0.09 | 0.565 | -0.04 | | -0.11 – 0.03 | 0.221 | -0.03 | | -0.10 – 0.03 | 0.346 |
| **Laptop** | 1.28 | 0.95 – 1.72 | 0.101 | -0.01 | | -0.07 – 0.05 | 0.784 | -0.09 | | -0.15 – -0.02 | 0.015 | 0 | | -0.07 – 0.07 | 0.951 | -0.06 | | -0.13 – 0.01 | 0.071 |
| **iPod** | 0.96 | 0.59 – 1.55 | 0.853 | -0.03 | | -0.12 – 0.06 | 0.527 | 0.07 | | -0.04 – 0.17 | 0.21 | -0.03 | | -0.14 – 0.08 | 0.635 | -0.12 | | -0.21 – -0.02 | 0.02 |
| **Smart watch** | 1.13 | 0.58 – 2.23 | 0.719 | 0 | | -0.14 – 0.13 | 0.945 | -0.01 | | -0.16 – 0.14 | 0.919 | -0.01 | | -0.17 – 0.15 | 0.894 | -0.06 | | -0.20 – 0.09 | 0.434 |
| **Pre-smartphone health measure** | 7.18 | 5.24 – 9.84 | <0.001 | 0.44 | | 0.40 – 0.47 | <0.001 | 0.33 | | 0.29 – 0.36 | <0.001 | 0.12 | | 0.09 – 0.16 | <0.001 | 0.33 | | 0.29 – 0.36 | <0.001 |

^a^The variables age, household income, parent education, parental monitoring, ownership of other devices are from year 3 follow-up
^b^Race and ethnicity data were reported by each youth participant’s caregiver.
^c^Income is expressed as the mean of the values from 10 self-reported income brackets: (1) <$5000; (2) $5000 to 11,999; (3) $12,000 to 15,999; (4) $16,000 to 24,999; (5) $25,000 to 34,999; (6) $35,000 to 49,999; (7) $50,000 to 74,999; (8) $75,000 to 99,999; (9) $100,000 to 199,999; and (10) over $200,000.
^d^Parental education represents the highest level of education attained by either parent.
^e^The variable pubertal stage is from the year 2 follow-up.

**eSupplementary Table 6-** Mediation analysis of associations between first smartphone acquisition and total mental health symptoms through digital exposure measures at age 13 (N = 2743).

| **Effect (DV ~ IV)** | **Unstandardized estimate** | **Standardized estimate** | **se** | **z** | **P value** | **CI lower** | **CI upper** |
| --- | --- | --- | --- | --- | --- | --- | --- |
| **Bedtime Streaming and Videogaming ~ Smartphone Ownership** | 0.295 | 0.148 | 0.04 | 7.68 | <0.001 | 0.22 | 0.37 |
| **Total BPM T Score ~ Bedtime Streaming and Videogaming** | 0.409 | 0.074 | 0.12 | 3.43 | <0.001 | 0.17 | 0.63 |
| **Total BPM T Score ~ Smartphone Ownership** | 0.141 | 0.013 | 0.23 | 0.62 | 0.53 | -0.32 | 0.55 |
| **Total BPM T Score ~ Total Screentime** | 0.753 | 0.131 | 0.13 | 5.87 | <0.001 | 0.50 | 1.00 |
| **Total BPM T Score ~ Having a Social Media Account** | 0.933 | 0.085 | 0.22 | 4.30 | <0.001 | 0.53 | 1.36 |
| **Indirect effect of Bedtime Streaming and Videogaming** | 0.120 | 0.011 | 0.04 | 3.07 | 0.002 | 0.05 | 0.21 |
| **Indirect effect of Total Screentime** | 0.19865827 | 0.018 | 0.04 | 4.61 | <0.001 | 0.12 | 0.29 |
| **indirect_Having a Social Media Account** | 0.253374551 | 0.023 | 0.06 | 4.17 | <0.001 | 0.14 | 0.37 |
| **Total Screentime ~ Smartphone Ownership** | 0.263729723 | 0.138 | 0.03 | 7.58 | <0.001 | 0.20 | 0.33 |
| **Having a Social Media Account~ Smartphone Ownership** | 0.271625513 | 0.271 | 0.02 | 14.93 | <0.001 | 0.23 | 0.31 |
| **Total Effect** | 0.713009293 | 0.065 | 0.22 | 3.30 | 0.001 | 0.28 | 1.12 |
| **Total Indirect Effect** | 0.572427772 | 0.052 | 0.08 | 6.93 | <0.001 | 0.41 | 0.73 |

BPM T-scores are norm-referenced measures of symptom severity, with higher scores indicating greater symptom severity.

Unstandardized estimates are reported on the original measurement scale of the variables. Standardized estimates are fully standardized coefficients (std.all), expressed in standard deviation units to facilitate comparison of effect sizes across variables. BPM outcomes were modeled using their original T-score metric; standardized estimates reflect coefficient standardization and do not represent BPM T-scores.

**eSupplementary Figure 1-** Distribution of Continuous Variables Included in Primary Analyses

**eSupplementary Figure 2-** Intercorrelations Among BPM Total, Internalizing, and Attention T-Scores

**eSupplementary Figure 3-** Intercorrelations Among Significant Screen Use Variables Considered for Mediation Analysis

**eSupplementary Figure 4-** Sensitivity Analysis Using Dichotomized Mental Health Outcomes

**eSupplementary Figure 5-** Sensitivity Analysis Using Dichotomized Digital Exposome Outcomes

**eSupplementary Figure 6-** Sensitivity Analysis Using Dichotomized Bedtime Routine Outcomes

**eSupplementary Figure 7-** Sensitivity Analysis Using Dichotomized Social and Behavioral Outcomes
